# AIM2 deletion enhances blood‐brain barrier integrity in experimental ischemic stroke

**DOI:** 10.1111/cns.13699

**Published:** 2021-06-22

**Authors:** Si‐yi Xu, Hui‐jie Bian, Shu Shu, Sheng‐nan Xia, Yue Gu, Mei‐juan Zhang, Yun Xu, Xiang Cao

**Affiliations:** ^1^ Department of Neurology Drum Tower Hospital Medical School and The State Key Laboratory of Pharmaceutical Biotechnology Institute of Brain Science Nanjing University Nanjing China; ^2^ Department of Neurology Nanjing Drum Tower Hospital Clinical College of Nanjing Medical University Nanjing China; ^3^ Department of Neurology Nanjing Drum Tower Hospital Clinical College of Traditional Chinese and Western Medicine Nanjing University of Chinese Medicine Nanjing China; ^4^ Jiangsu Key Laboratory for Molecular Medicine Medical School of Nanjing University Nanjing China; ^5^ Jiangsu Province Stroke Center for Diagnosis and Therapy Nanjing China

**Keywords:** AIM2, blood‐brain barrier, endothelial cells, ischemic stroke, STAT3

## Abstract

**Aims:**

Ischemic stroke is a life‐threatening disease with limited therapeutic strategies. Blood‐brain barrier (BBB) disruption is a critical pathological process that contributes to poor outcomes in ischemic stroke. We previously showed that the microglial inhibition of the inflammasome sensor absent in melanoma 2 (AIM2) suppressed the inflammatory response and protected against ischemic stroke. However, whether AIM2 is involved in BBB disruption during cerebral ischemia is unknown.

**Methods:**

Middle cerebral artery occlusion (MCAO) and oxygen‐glucose deprivation/reoxygenation (OGD/R) were used to mimic cerebral ischemia in mice and brain microvascular endothelial cells (HBMECs), respectively. The infarct volume, neurological deficits, and BBB permeability were measured in mice after MCAO. Transendothelial electrical resistance (TEER) and neutrophil adhesion to the HBMEC monolayer were assessed after OGD/R treatment. Western blot and immunofluorescence analyses were conducted to evaluate the expression of related proteins.

**Results:**

AIM2 was shown to be expressed in brain endothelial cells and upregulated after ischemic stroke in the mouse brain. AIM2 deletion reduced the infarct volume, improved neurological and motor functions, and decreased BBB disruption. In vitro, OGD/R significantly increased the protein levels of AIM2 and ICAM‐1 and decreased those of the tight junction (TJ) proteins ZO‐1 and occludin. AIM2 knockdown effectively protected BBB integrity by promoting the expression of TJ proteins and decreasing ICAM‐1 expression and neutrophil adhesion. Mechanistically, AIM2 knockdown reversed the OGD/R‐induced increases in ICAM‐1 expression and STAT3 phosphorylation in brain endothelial cells. Furthermore, treatment with the p‐STAT3 inhibitor AG490 mitigated the effect of AIM2 on BBB breakdown.

**Conclusion:**

Our findings indicated that inhibiting AIM2 preserved the BBB integrity after ischemic stroke, at least partially by modulating STAT3 activation and that AIM2 may be a promising therapeutic target for cerebral ischemic stroke.

## Graphical abstract

AIM2 deletion in brain endothelial cells reversed ischemia‐induced BBB injury by suppressing ICAM‐1 expression and neutrophil adhesion in a STAT3 signaling‐ dependent manner.

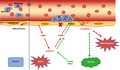

## 1 INTRODUCTION

Stroke is a life‐threatening disease that leads to long‐term disability and mortality worldwide, and approximately 85% of all strokes are classified as ischemic. Currently, tissue plasminogen activator (tPA) is the only FDA‐approved therapeutic agent for ischemic stroke. However, few patients can benefit from this treatment because its clinical use is limited by the increased risk of intracerebral bleeding complications and narrow therapeutic time window.^1^ Therefore, new therapies for ischemic stroke are desperately needed.

The blood‐brain barrier (BBB) is a structure composed of cerebral endothelial cells, pericytes, extracellular matrix, and astrocytic endfeet that maintains the microenvironment of the central nervous system (CNS). During cerebral ischemia, the permeability of the BBB increases, and its stability declines.^2^ Clinically, the degree of BBB damage is usually associated with reduced survival rates in patients with acute ischemic stroke.^3^ Our previous study demonstrated that treatment with sodium tanshinone IIA sulfonate can improve the neurological functional outcomes of patients with acute ischemic stroke by attenuating BBB breakdown.^4^ Similarly, BBB disruption is exacerbated after reperfusion in mouse and rat models of middle cerebral artery occlusion (MCAO).^5^, ^6^ Recently, extensive studies have suggested that the inflammatory response is the major cause of BBB breakdown after cerebral ischemia. Inflammasomes are one of the most crucial components of the innate immune system and play important roles in the pathogeneses of various inflammation‐related neurological diseases.^7^ tPA was reported to promote inflammasome activation after ischemic stroke in hyperglycemic mice.^8^ Inflammasome activation was shown to lead to BBB disruption, hemorrhagic transformation, and poor outcomes in hyperglycemic patients suffering from ischemic stroke.^9^ Inhibition of caspase‐1, a core inflammasome complex component, might ameliorate ischemia‐associated BBB dysfunction and loss of integrity.^10^ Although these findings indicate that inflammasomes are associated with ischemia‐reperfusion injury and BBB damage, the detailed mechanisms remain unknown.

Absent in melanoma 2 (AIM2), an important inflammasome component, was initially identified in cancer‐associated studies and has been reported to suppress many types of cancer.^11^ AIM2 can recruit apoptosis speck‐like protein (ASC) and caspase‐1 to form a multiprotein complex to support the maturation and secretion of interleukin (IL)‐1β and IL‐18. This multimeric protein complex is also called the AIM2 inflammasome and has been demonstrated to be a critical mediator of the neuroinflammatory response during ischemic stroke.^12^, ^13^ A very recent study showed that AIM2 was primarily localized in microglial and endothelial cells in mice with poststroke cognitive impairment.^14^ Additionally, our previous study revealed that a selective inhibitor of histone deacetylase 3 (HDAC3) alleviated the inflammatory response and protected against ischemic stroke by suppressing the activation of the AIM2 inflammasome in microglia.^15^ Despite these findings, a detailed characterization of the role of AIM2 in BBB damage, especially in endothelial cells, after ischemic stroke remains elusive.

Considering the above factors, we aimed to explore the effects of AIM2 on the BBB integrity in mice with MCAO‐induced injury and in oxygen‐glucose deprivation/reoxygenation (OGD/R)‐induced human brain microvascular endothelial cells (HBMECs). Furthermore, we aimed to elucidate the mechanism underlying the effects of AIM2 on BBB disruption. These results should further our understanding of the AIM2 inflammasome and provide a therapeutic strategy for BBB impairment after cerebral ischemia.

## 2 METHODS

### 2.1 Experimental animals

AIM2 knockout (AIM2^−/−^) mice were generated using CRISPR/Cas9 technology and were purchased from the Model Animal Research Center of Nanjing University (Nanjing, China). Age‐matched C57BL/6J littermate mice were used as controls. Experiments were carried out on eight‐week‐old male mice with an average weight of 22–25 g. These mice were housed 6 per cage on a light‐dark cycle of 12 h, with access to adequate food and water supplies, and under suitable temperature (22±2℃) and humidity (55±5%) conditions. All procedures were approved by the Animal Care Committee of Nanjing University and followed the ARRIVE guidelines.^16^

### 2.2 Cell culture

HBMECs were obtained from ScienCell Research Laboratories and cultured in endothelial cell medium (ECM, ScienCell, CA, USA) in a humidified atmosphere of 5% CO_2_ at 37℃. All HBMECs used in vitro were passaged fewer than 10 times. Primary brain microvascular endothelial cells (PBMECs) were isolated from the cortices of wild‐type (WT) and AIM2^−/−^ mice and cultured as we previously described.^17^ Briefly, the cortical tissues were cut into small pieces and digested with 0.2% collagenase/dispase containing 20 U/ml DNase I. The PBMECs were cultured in ECM supplemented with 10% fetal bovine serum (FBS, Gibco, CA, USA) in gelatin‐coated plastic culture flasks. The medium was changed every 2–3 days, and the cells were passaged no more than 3 times before use.

### 2.3 MCAO model mice

In brief, mice were anesthetized with 1% pentobarbital sodium (45 mg/kg i.p.) after being weighed. A silicon‐coated 6/0 monofilament nylon suture (Doccol Corporation, MA, USA) was inserted into the beginning of the middle cerebral artery (MCA) through the internal carotid artery until the ipsilateral blood flow of the MCA supply territory decreased to less than 20% of baseline as monitored by laser Doppler flowmetry (Perimed Corporation, Stockholm, Sweden). After 1 h of ischemia, blood reperfusion was achieved by withdrawing the suture. Sham‐operated mice underwent the same procedures without MCA occlusion. An electric blanket was used during the operation to maintain the body temperatures of the mice at 36–38℃.

### 2.4 Behavioral testing

All behavioral testing was performed in a double‐blinded manner. A neurological severity score (NSS) was adopted to evaluate the neurological function of the mice. The NSS included data for a composite analysis of sensory function, motor function, reflexes, and balance which were graded on a scale of 0–18. Higher scores indicated more severe injury. The grip strength test was used to measure the maximum strength of the mice. In this test, a mouse was pulled by the tail toward the T‐bar of the machine (GS3, Bioseb, France). As the mouse grasped both sides of the T‐bar with both of its forelimbs, it was pulled back at a constant velocity until it was out of the machine. The test was performed five times per mouse, and the maximum peak force was recorded.

### 2.5 Measurement of infarct volume

To measure the infarct volume, the mouse brains were stained with 2% 2,3,5‐triphenyltetrazolium chloride (TTC, Sigma, St. Louis, MO, USA) 1 d and 3 d after MCAO. The brains were continuously sliced into six coronal (1‐mm thick) and were then immersed in TTC solution in the dark at 37℃ for 15 min. The slices were fixed with 4% paraformaldehyde, photographed, and analyzed with ImageJ software (ImageJ 1.5, NIH, USA). After correcting for edema, we calculated the infarct volume as a percentage of the total brain volume.

### 2.6 Measurement of Evans Blue (EB) dye extravasation

BBB permeability was evaluated by measuring EB dye extravasation. EB dye (2% in saline, 4 ml/kg) was injected into mice via the tail vein 1 d after MCAO. Five hours later, the mice were anesthetized and perfused with cold 0.9% NaCl through the coronary artery. To quantify EB leakage, the brains were cut into slices for analysis. The hemispheres were weighed and immersed in formamide (10 ml/kg, Sigma) at 60℃ for 24 h. The samples were centrifuged (5000 rpm, 10 min) at 4℃, and the absorbance of the supernatants was then measured at 620 nm in a spectrophotometer (Tecan Trading AG, Switzerland). The results were compared with a standard curve and are shown as micrograms of EB per gram of brain tissue.

### 2.7 OGD/R treatment

The OGD/R model was used to mimic ischemia in vitro. HBMECs were seeded into a 12‐well plate and cultured in complete medium containing glucose. When the cells were confluent at the bottom of the well, the complete medium was replaced with glucose‐free DMEM. The plate was placed in a hypoxia chamber (Billups‐Rothenberg, Del Mar, CA, USA) that contained 95% N_2_/5% CO_2_ for 4 h. Reoxygenation was initiated by adding complete medium, and the cells were incubated in a normoxic incubator (95% O_2_/5% CO_2_, 37℃) for another 20 h. PBMECs exposed to OGD/R (2 h OGD/20 h reperfusion) as previously described.^18^ AG490 was used to inhibit STAT3 pathway activity. Cells were treated with 40 mM AG490 for 2 h before OGD/R treatment and throughout all the experimental periods.

### 2.8 Western blot analysis

Protein lysates were prepared from mouse brain samples and treated HBMECs using RIPA buffer (Thermo, Waltham, MA, USA) containing phosphatase and protease inhibitors. To separate the proteins by size, the prepared protein lysates were loaded onto an SDS‐PAGE. The proteins were transferred to PVDF membranes, and the membranes were then incubated with primary antibodies specific for AIM2 (Santa Cruz Biotechnology, sc‐515514, 1:500), ZO‐1(Invitrogen, 40–2200, 1:1000), occludin (ProteinTech, 13409–1‐AP, 1:1000), STAT3 (Cell Signaling Technology, 9139S, 1:1000), phospho‐STAT3 (p‐STAT3, Cell Signaling Technology, 9145S, 1:1000), myeloperoxidase (MPO, Abcam, ab208670, 1:1000), ICAM‐1 (Invitrogen, MA5407, 1:500), and GAPDH (BioWorld, AP0063, 1:4000) at 4℃ overnight. After the membranes were washed with TBST buffer, horseradish peroxidase (HRP)‐conjugated anti‐mouse or anti‐rabbit (Bioworld, 1:2000) secondary antibodies were added and incubated at room temperature for another 2 h. Bands were visualized in a Gel‐Pro system (Tanon Technologies, Shanghai, China), and band intensities were quantified with ImageJ software.

### 2.9 Immunofluorescence staining

Immunofluorescence staining was performed according to a previously described protocol.^15^ In brief, brain sections or cells were fixed with 4% paraformaldehyde. After permeabilization and blocking, the samples were incubated at 4℃ overnight with primary antibodies specific for the following proteins: AIM2 (Santa Cruz Biotechnology, sc‐515514, 1:200), CD31 (Santa Cruz Biotechnology, sc‐18916, 1:200), ZO‐1(ProteinTech, 21773–1‐AP, 1:300), occludin (Invitrogen, 33–1500, 1:100), p‐STAT3 (Cell Signaling Technology, 9145S, 1:200), and mouse IgG (BioWorld, BD0050, 1:500). Subsequently, the samples were incubated with the appropriate fluorescence‐conjugated secondary antibodies for 2 h at room temperature in the dark. DAPI (1:1000) was used to stain cell nuclei. Images were screened using a fluorescence microscope (Olympus IX73, Japan) or confocal laser‐scanning microscope (Olympus FV3000, Japan).

### 2.10 Real‐time PCR

Total RNA was extracted from cells with TRIzol reagent (Invitrogen, CA, USA) and reverse transcribed into cDNA by using a PrimeScript RT Reagent Kit (Takara, Dalian, China). Finally, quantitative PCR was performed in an ABI StepOne Plus PCR instrument (Applied Biosystems, CA, USA) with a SYBR green kit (Applied Biosystems), and the relative gene expression levels were normalized to that of GAPDH. The primer sequences were as follows: AIM2 F: TTGAGACCCAAGAAGGCAAG, R: CGTGAGGCGCTATTTACCTC; and GAPDH F: GCCAAGGCTGTGGGCAAGGT, R: TCTCCAGGCGGCACGTCAGA.

### 2.11 Measurement of transendothelial electrical resistance (TEER)

The TEER of cultured HBMEC monolayers was measured by using an epithelial voltohmmeter (EVOM, World Precision Instruments, USA) according to the manufacturer's instructions. The experiment was performed three times, and the mean value was recorded. We used an empty Transwell chamber without any cells as the blank control.

### 2.12 Assessment of neutrophil adhesion to HBMECs

Mouse bone marrow neutrophils were used for this experiment. In brief, bone marrow cells were flushed from the femurs and tibias of mice. Erythrocytes were lysed with ACK lysis buffer (Gibco), and the tissue suspension was centrifuged at 1500 rpm for 5 min. After washing with sterile PBS, the collected cells were stained with a PE‐conjugated anti‐Ly6G antibody (Invitrogen, 12–9668–82) and then labeled with magnetic anti‐PE microbeads (BD Biosciences, Carlsbad, CA, USA). Subsequently, the cells were separated using a MACS column and MACS separator according to the manufacturer's instructions. The neutrophil purity exceeded 95% as determined by flow cytometry.

Isolated neutrophils were cultured in Dulbecco's modified Eagle medium (DMEM, Gibco) supplemented with 10% FBS and labeled with 10 μM DiI dye (Beyotime Biotech, Nantong, China) at 37℃ for 20 min. After washing three times with PBS, fluorescently labeled neutrophils were added to the HBMEC monolayer (neutrophil: HBMEC ratio =1:10) and allowed to adhere for 30 min at 37℃ in a humidified 5% CO_2_ incubator. The nonadherent neutrophils were removed by thoroughly washing with PBS, and the number of adherent neutrophils was determined by fluorescence microscopy (excitation at 549 nm, emission at 565 nm).

### 2.13 Transfection experiments

The lentivirus expressing AIM2 siRNA (Lv‐siAIM2) and control lentivirus (Lv‐control) were generated by GeneChem (Shanghai, China). According to the manufacturer's instructions, sparse HBMECs were incubated with Lv‐siAIM2 or Lv‐control (MOI =20) for 1 d, after which the medium was replaced with fresh culture medium and the cells were incubated for an additional 3 d. The expression of AIM2 was determined by Western blot and real‐time PCR.

### 2.14 Statistical analysis

All data were analyzed using SPSS 18.0 software (IBM Corp, Armonk, NY, USA) and are expressed as the mean ±standard error of the mean (SEM) values. The normality of the data distribution was analyzed by the Shapiro‐Wilk test. To compare differences between two groups, normally distributed continuous variables were compared by Student's t test, while non‐normally distributed variables were compared by the Mann‐Whitney test. For multiple comparisons among three groups or more groups, data were analyzed using one‐way analysis of variance (ANOVA) followed by Bonferroni's post hoc test if the data were normally distributed or by the Kruskal‐Wallis test if the data were non‐normally distributed. A *p* value of <0.05 was considered to indicate a statistically significant difference between experimental results.

## 3 RESULTS

### 3.1 AIM2 was upregulated in endothelial cells after experimental ischemic stroke

**FIGURE 1:**
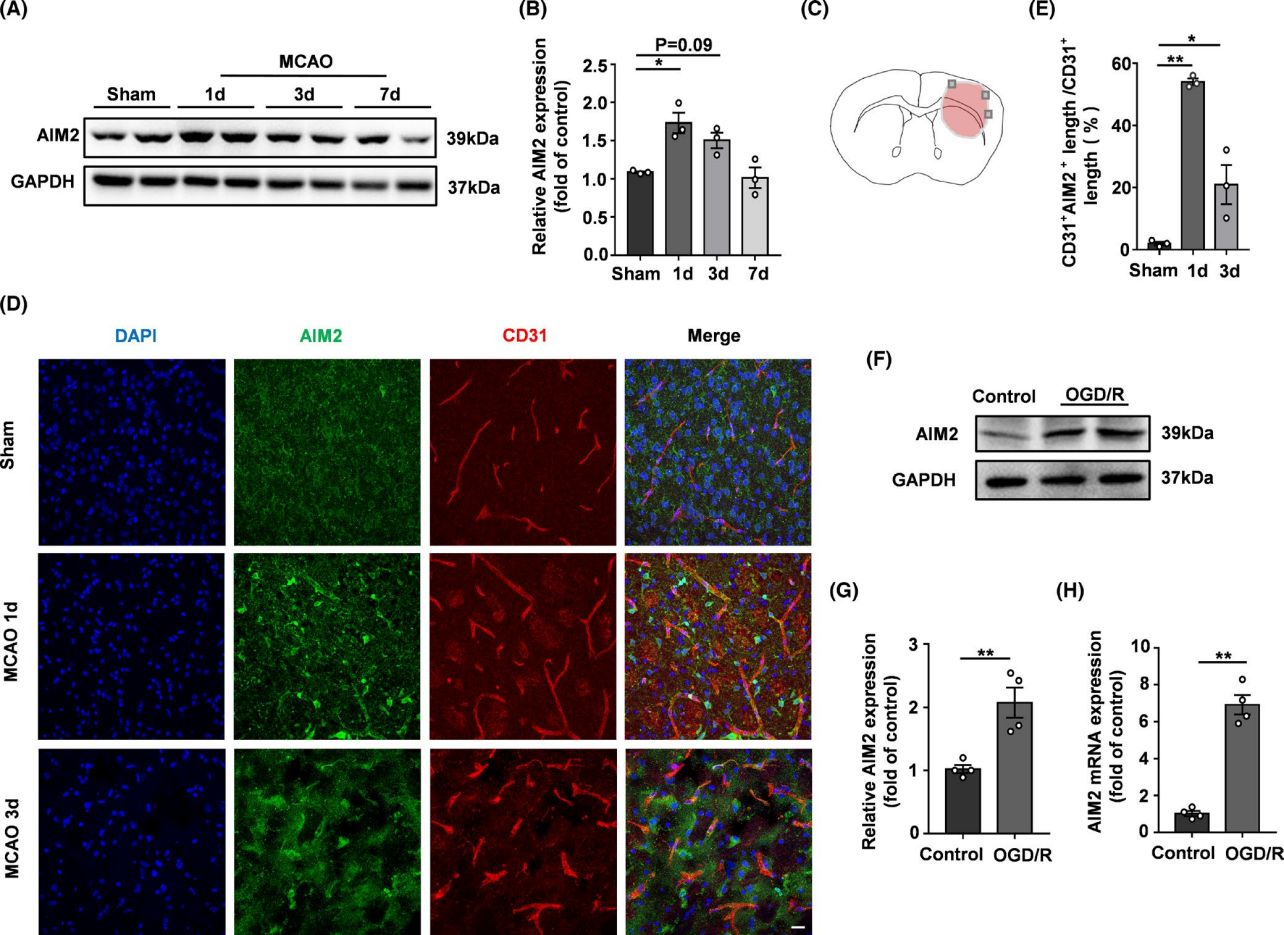
AIM2 was expressed in brain endothelial cells, and its expression increased after ischemic stroke. A, Western blot images of AIM2 and GAPDH expression on days 1, 3, and 7 after MCAO. B, Quantification of AIM2 expression normalized to that of GAPDH. C, Image showing the location at which immunostaining was conducted in the penumbra of the infarcted brain. D, Immunofluorescence images of DAPI (blue)/AIM2 (green)/CD31 (red) colocalization on days 1 and 3 after MCAO. Scale bar: 20 μm. E, Percentage of the CD31^+^AIM2^+^ vascular length with respect to the total CD31^+^ vascular length. F, Western blot images of AIM2 and GAPDH expression after OGD/R. G, Quantification of AIM2 expression normalized to that of GAPDH. H, AIM2 mRNA expression after OGD/R. All data are presented as the mean ±SEM. *p < 0.05 and ** *p* < 0.01 compared with the control group

To verify whether the AIM2 expression level changed after ischemic stroke, we first analyzed AIM2 expression in the brains of mice after MCAO by Western blot. The AIM2 protein level was obviously increased on the infarcted side of the brain cortex on day 1 after MCAO (Figure 1A and B). No significant differences were observed on days 3 and 7, but an increasing trend in AIM2 expression was observed on day 3 after MCAO. To determine whether AIM2 is associated with BBB breakdown after MCAO, we immunofluorescently labeled AIM2 and the endothelial cell marker CD31 in the ischemic penumbra (Figure 1C). AIM2 colocalized with CD31, and the percentage of the CD31^+^AIM2^+^ vascular length with respect to the total CD31^+^ vascular length was dramatically increased on day 1 after MCAO (Figure 1D and E).

An endothelial cell monolayer was used as an in vitro BBB model, and the OGD/R model was established to mimic in vivo ischemia/reperfusion injury. The expression of AIM2 was analyzed by Western blot and qPCR. After 4 h of OGD treatment followed by 20 h of reoxygenation, AIM2 expression was increased significantly (Figure 1F‐H), which was consistent with the in vivo experimental results. Based on the results of the in vivo and in vitro experiments, we next investigated whether AIM2 deletion benefits BBB integrity after ischemic stroke.

### 3.2 AIM2 knockout reduced the neurofunctional deficits in mice after MCAO

**FIGURE 2:**
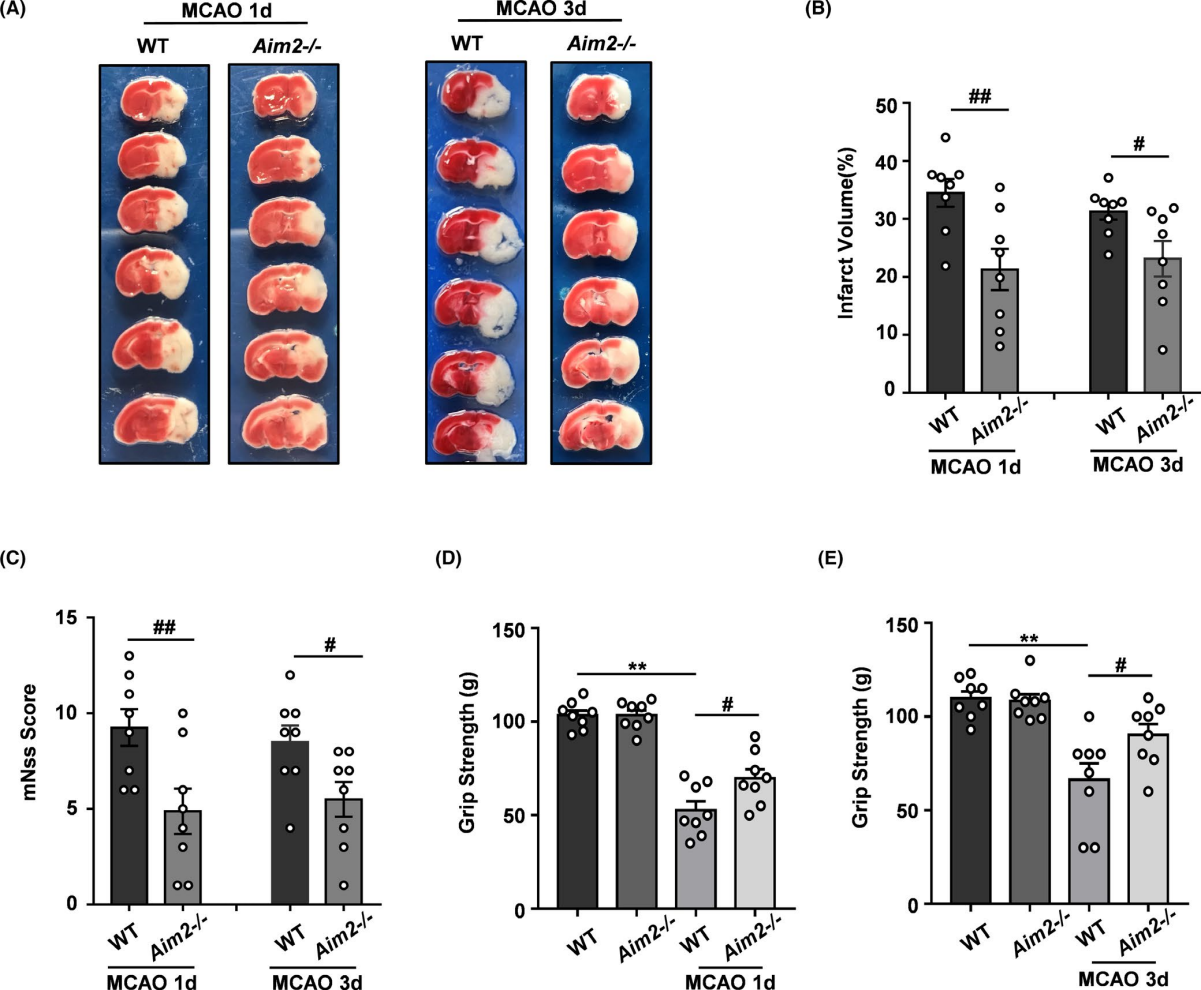
AIM2 knockout reduced the infarct volume and improved neurological outcomes after ischemic stroke. A, Representative brain sections stained with TTC on days 1 and 3 after MCAO. B, Infarct volume (n=8). C, Modified NSS score (n=8). D, Grip strength on day 1(n=8). E, Grip strength on day 3(n=8). All data are presented as the mean ±SEM. **p < 0.01 compared with the WT group. ^#^ *p* < 0.05 and ^##^ *p* < 0.01 compared with the MCAO‐WT group

To determine whether AIM2 knockout could protect mice against ischemic injury after MCAO, we used AIM2^−/−^mice generated by CRISPR/Cas9 gene editing. We selected days 1 and 3 after MCAO as the time points for the following experiments. The infarct size was evaluated by TTC staining, revealing size reductions in approximately 14.03% and 8.16% in the AIM2^−/−^ group compared with the WT group at 1 and 3 days after MCAO, respectively (Figure 2A and B). Neurological performance assessments, such as evaluations of the NSS and grip strength, were used. The NSS revealed a significant genotypic difference, showing that AIM2^−/−^ mice had lower scores than WT mice (Figure 2C). The grip strength was not significantly different between the two groups before the MCAO procedure (baseline), while the AIM2^−/−^ group showed obviously greater strength than the WT group after MCAO (Figure 2D and E), consistent with the NSS scores. These results demonstrated that AIM2 deletion ameliorated ischemic brain injury.

### 3.3 AIM2 knockout partially rescued the BBB breakdown in mice after MCAO

**FIGURE 3:**
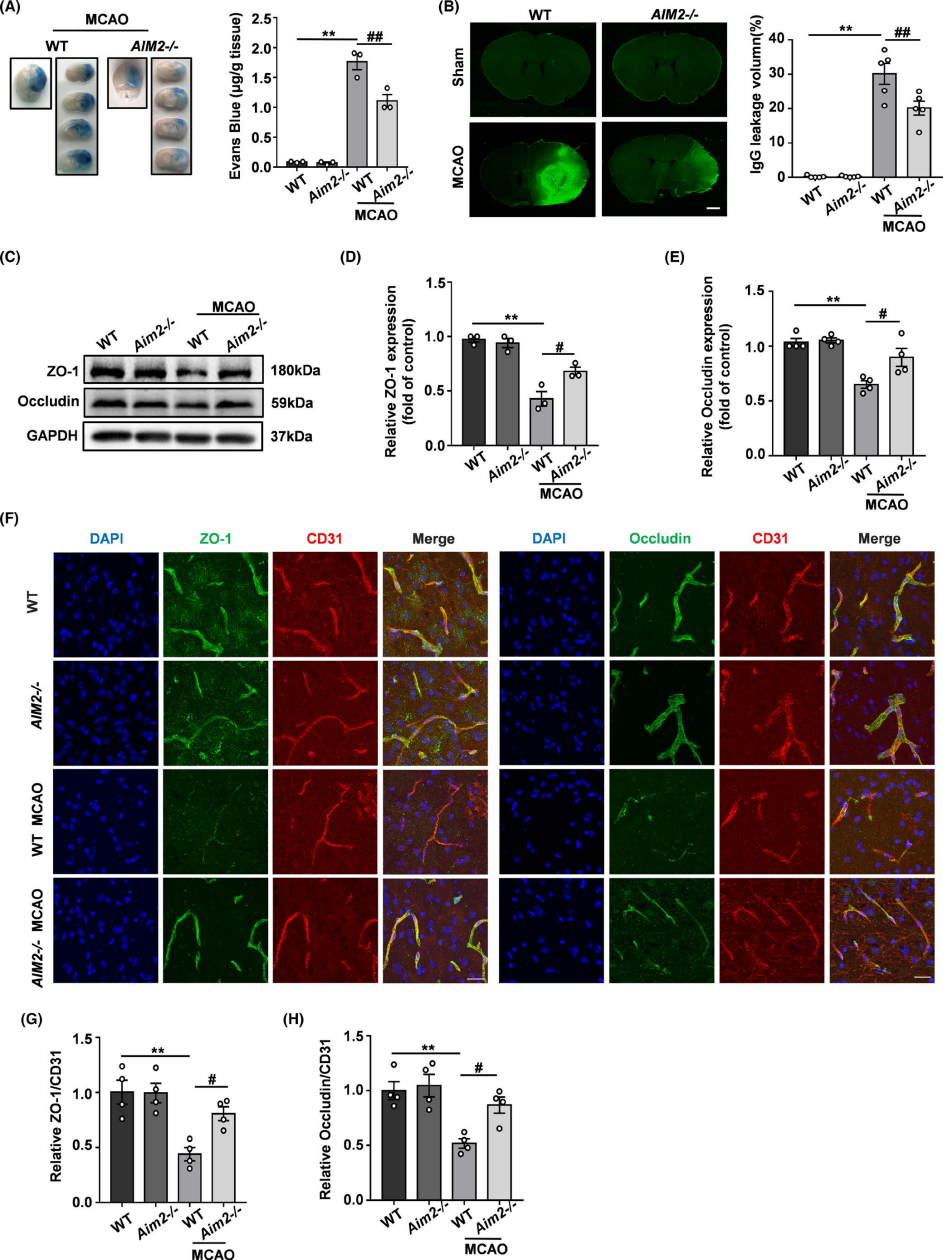
AIM2 knockout alleviated BBB breakdown after ischemic stroke. A, EB dye (n=3) and B, IgG extravasation (n=5) in the WT, AIM2^−/−^, MCAO‐WT, and MCAO‐AIM2^−/−^ groups on day 1 after MCAO. Scale bar: 1 mm. C, Western blot analysis of ZO‐1, occludin, and GAPDH on day 1 after MCAO. D, E, Quantification of ZO‐1 and occludin expression normalized to that of GAPDH. F, Representative immunofluorescence images of DAPI (blue)/ZO‐1 (green) and occludin (green)/CD31 (red) colocalization on day 1 after MCAO. Scale bar: 20 μm. G, Quantification of the immunofluorescence intensity of ZO‐1 with respect to that of CD31. H, Quantification of the immunofluorescence intensity of occludin with respect to that of CD31. All data are presented as the mean ±SEM. ** *p* < 0.01 compared with the WT group. ^#^ *p* < 0.05 and ^##^ *p* < 0.01 compared with the MCAO‐WT group

Next, we evaluated BBB leakage by performing EB extravasation and IgG immunostaining assays. While the BBB was intact in the sham‐operated groups, obvious increases in EB extravasation and IgG leakage were observed in the MCAO groups on day 1 after the MCAO procedure. The BBB integrity of AIM2^−/−^ mice was better than that of WT mice after MCAO, as evidenced by significant decreases in EB signals and IgG leakage (Figure 3A and B). The BBB is composed mainly of endothelial cells connected by tight junctions (TJs) and TJ proteins, such as ZO‐1 and occludin, which play critical roles in BBB integrity. Therefore, we measured the expression of these TJ proteins by Western blot and immunofluorescence analyses. ZO‐1 and occludin expression was significantly decreased on day 1 after MCAO, indicating that the BBB integrity was disrupted after ischemic stroke. However, AIM2 knockout rescued the expression of these TJ proteins after MCAO (Figure 3C‐E). The immunofluorescence staining results indicated that the colocalization of TJ proteins with the endothelial marker CD31 was markedly decreased after MCAO. However, this colocalization was obviously stronger in the AIM2^−/−^group than in the WT group after MCAO, consistent with the Western blot results (Figure 3F‐H). These results suggested that AIM2 knockout preserves the BBB integrity after MCAO.

### 3.4 AIM2 knockdown alleviated BBB breakdown after OGD/R treatment in vitro

**FIGURE 4:**
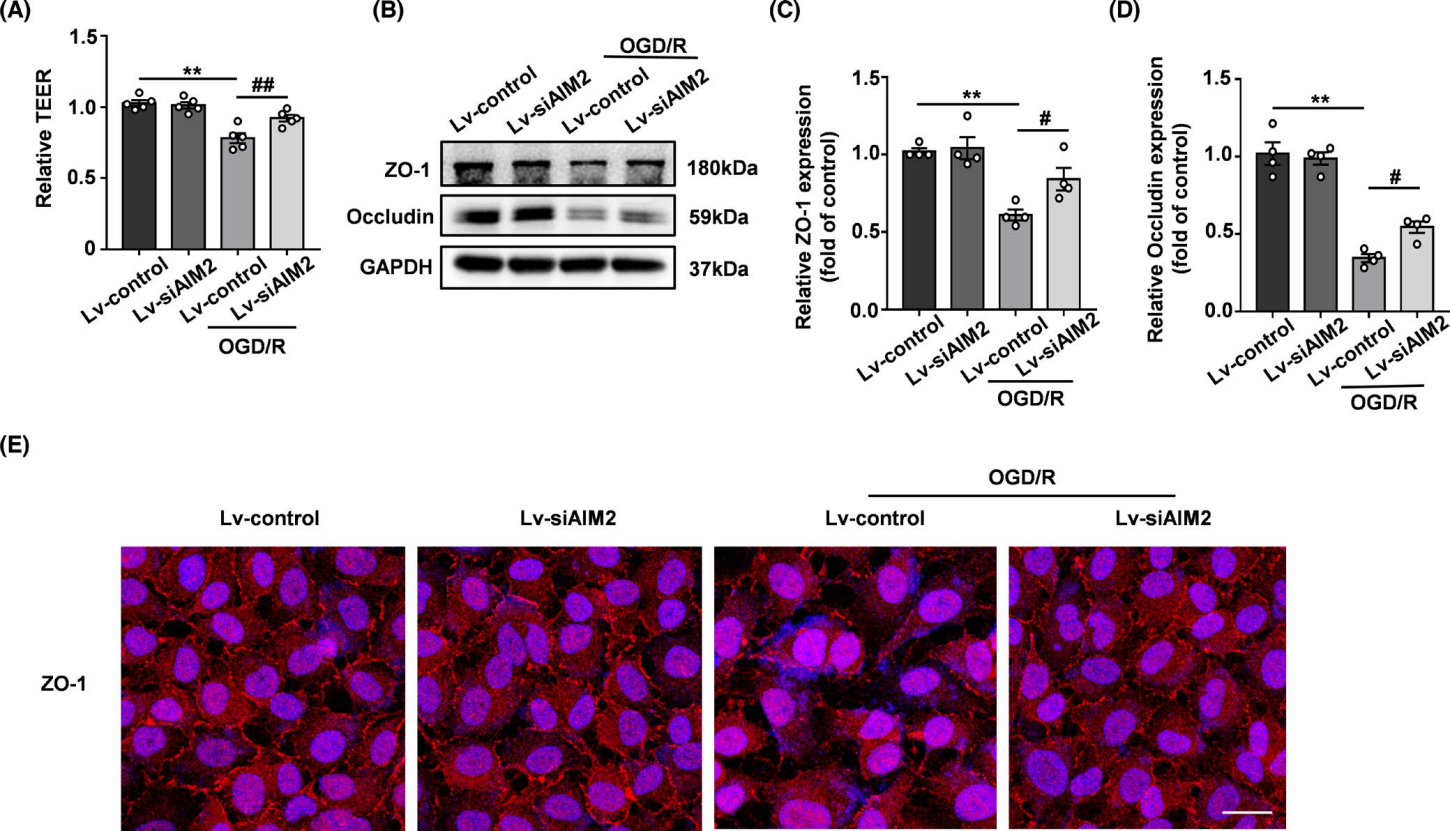
Silencing AIM2 rescued BBB breakdown after OGD/R in vitro. A, Quantification of transendothelial permeability as determined by the TEER assay (n=5). B, Western blot analysis of ZO‐1, occludin, and GAPDH after OGD/R. C, D, Quantification of the protein levels of ZO‐1 and occludin normalized to those of GAPDH. E, Representative immunofluorescence images of DAPI (blue)/ZO‐1 (red) colocalization after OGD/R. Scale bar: 20 μm. All data are presented as the mean ±SEM. ** *p* < 0.01 compared with the control group. ^#^ *p* < 0.05 and ^##^ *p* < 0.01 compared with the OGD/R‐control group

AIM2 expression was silenced in endothelial cells in vitro with a lentivirus. The transduction efficiencies of Lv‐siAIM2 and Lv‐control were assessed by immunofluorescence, Western blot, and qPCR analyses, revealing that AIM2 was successfully knocked down (Figure S1A‐D). TEER assays were used to evaluate the BBB integrity in vitro. As shown in Figure 4A, the TEER was significantly decreased after OGD/R, but was higher in the Lv‐siAIM2 group than in the Lv‐control group after OGD/R. Furthermore, we assessed the levels of TJ proteins by Western blot and immunofluorescence analyses. Similar to the patterns observed in vivo, the TJ proteins levels were decreased after OGD/R, whereas AIM2 knockdown alleviated these decreases (Figure 4B‐E).

### 3.5 AIM2 deletion enhanced the BBB integrity in experimental ischemic stroke by decreasing ICAM‐1 expression via the STAT3 signaling pathway

**FIGURE 5:**
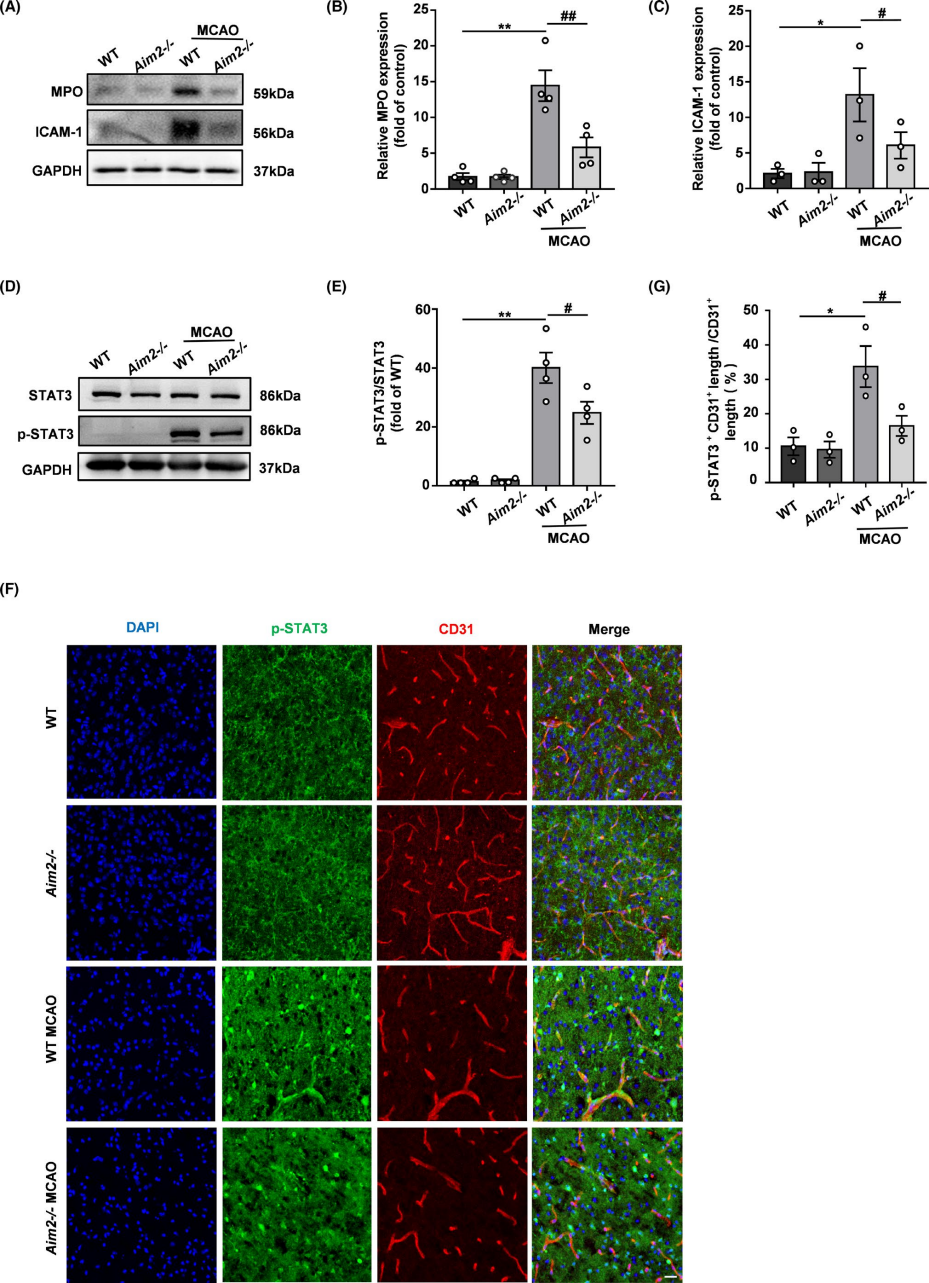
AIM2 knockout decreased neutrophil infiltration and adhesion after ischemic stroke, and these effects were potentially associated with the decreased p‐STAT3 expression in endothelial cells. A, The in vivo protein levels of MPO, ICAM‐1, and GAPDH on day 1 after MCAO as determined by Western blot. B, C, Quantification of the levels of MPO and ICAM‐1 normalized to that of GAPDH. D, The in vivo protein levels of STAT3, p‐STAT3, and GAPDH on day 1 after MCAO as determined by Western blot. E, Quantification of p‐STAT3. F, Immunofluorescence images of DAPI (blue)/p‐STAT3 (green)/CD31 (red) colocalization in the WT, AIM2^−/−^, MCAO‐WT, and MCAO‐AIM2^−/−^ groups on day 1 after MCAO. Scale bar: 20 μm. G, Percentage of the CD31^+^p‐STAT3^+^ vascular length with respect to the total CD31^+^ vascular length. All data are presented as the mean ±SEM. *p < 0.05 and ** *p* < 0.01 compared with the WT group. ^#^ *p* < 0.05 and ^##^ *p* < 0.01 compared with the MCAO‐WT group

As ICAM‐1 is an endothelial and leukocyte‐associated transmembrane protein, its expression is significantly increased under hypoxic conditions, which promotes neutrophil adhesion and BBB damage. MPO is an indicator of neutrophil infiltration into the brain. Thus, we evaluated the expression of ICAM‐1 and MPO in the brains of AIM2^−/−^ and WT mice after MCAO. As shown in Figure 5A‐C, relatively low basal expression levels of MPO and ICAM‐1 were found in the brain. Although these levels increased significantly after the induction of hypoxic ischemia, AIM2 knockout suppressed these increases. Because recent evidence suggests that STAT3 phosphorylation is dramatically upregulated in the brains of mice after MCAO, we investigated whether the protective effect of AIM2 deletion after MCAO is associated with the phosphorylation of STAT3. As shown in Figure 5D and E, the p‐STAT3 level did not significantly differ between the two groups before MCAO, while p‐STAT3 expression was obviously increased in both groups after MCAO. However, p‐STAT3 was expressed at a lower level in the AIM2^−/−^ group than in the WT group after MCAO. To further characterize whether the level of p‐STAT3 was changed in endothelial cells, double labeling of p‐STAT3 and CD31 was performed in the intact and ipsilateral penumbral areas of mouse brains after ischemic stroke. The colocalization of p‐STAT3 and CD31 was significantly increased in both groups after MCAO. However, this colocalization was less extensive in AIM2^−/−^ mice than in WT mice after MCAO (Figure 5F and G), suggesting that the protective effect of AIM2 deletion may be associated with a decrease in STAT3 phosphorylation in endothelial cells.

**FIGURE 6:**
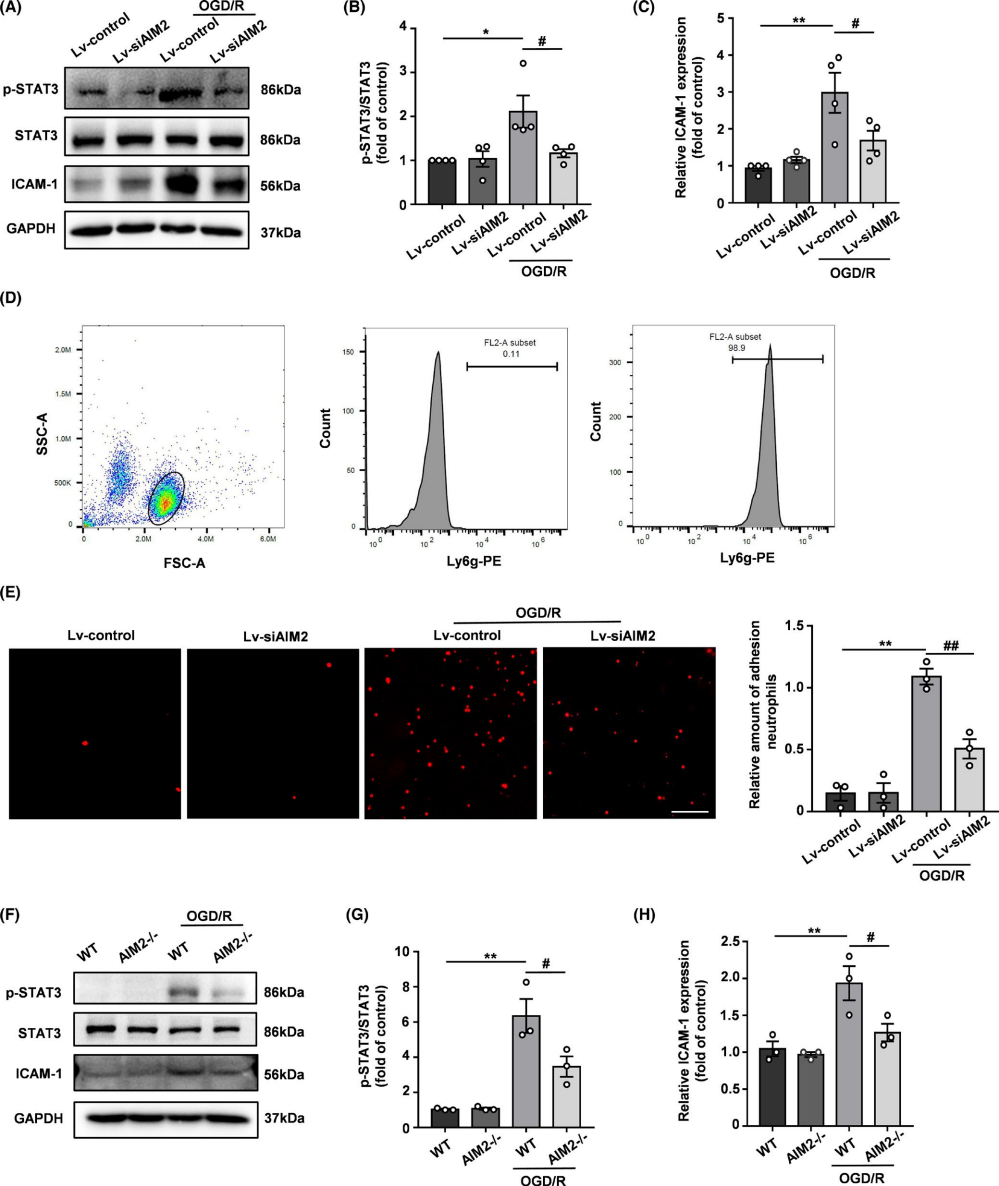
AIM2 knockdown decreased neutrophil adhesion in vitro after OGD/R, and this effect was potentially associated with a decrease in p‐STAT3 expression in endothelial cells. A, The protein levels of STAT3, p‐STAT3, ICAM‐1, and GAPDH in HBMECs after OGD/R as determined by Western blot. B, C, Quantification of the levels of p‐STAT3 and ICAM‐1 normalized to that of GAPDH. D, The neutrophil purity was examined by flow cytometry. E, Representative images and quantification of neutrophil adhesion to HBMEC monolayers in vitro. Scale bar: 100 μm. F, Western blot images of STAT3, p‐STAT3, ICAM‐1, and GAPDH expression in PBMECs after OGD/R. G, H, Quantification of the levels of p‐STAT3 and ICAM‐1 normalized to that of GAPDH. All data are presented as the mean ±SEM. * *p* < 0.05 and ** *p* < 0.01 compared with the Lv‐control group. ^#^ *p* < 0.05 and ^##^ *p* < 0.01 compared with the OGD/R‐Lv‐control group

Similarly, we verified this mechanism in vitro by analyzing the expression of ICAM‐1 and STAT3 signaling pathway by Western blot. As shown in Figure 6A‐C, OGD/R increased the levels of ICAM‐1 and p‐STAT3 in HBMECs, and AIM2 knockdown obviously alleviated these increases. To mimic neutrophil adhesion in vitro, an adhesion experiment with neutrophils was performed. We isolated mouse bone marrow neutrophils and evaluated their purity by flow cytometry, revealing a purity greater than 95% (Figure 6D). OGD/R significantly increased the adhesion of neutrophils to HBMECs compared with that in the control group, whereas AIM2 knockdown decreased the adhesion of neutrophils to HBMECs (Figure 6E). In addition, we isolated PBMECs from the cortices of WT and AIM2^−/−^ mice to further clarify the role of AIM2 in endothelial cells after OGD/R. In accordance with the results obtained with HBMECs, AIM2 deletion reduced the OGD/R‐induced expression of ICAM‐1 and p‐STAT3 in AIM2^−/−^ PBMECs compared with the WT group cells (Figure 6F‐H).

**FIGURE 7:**
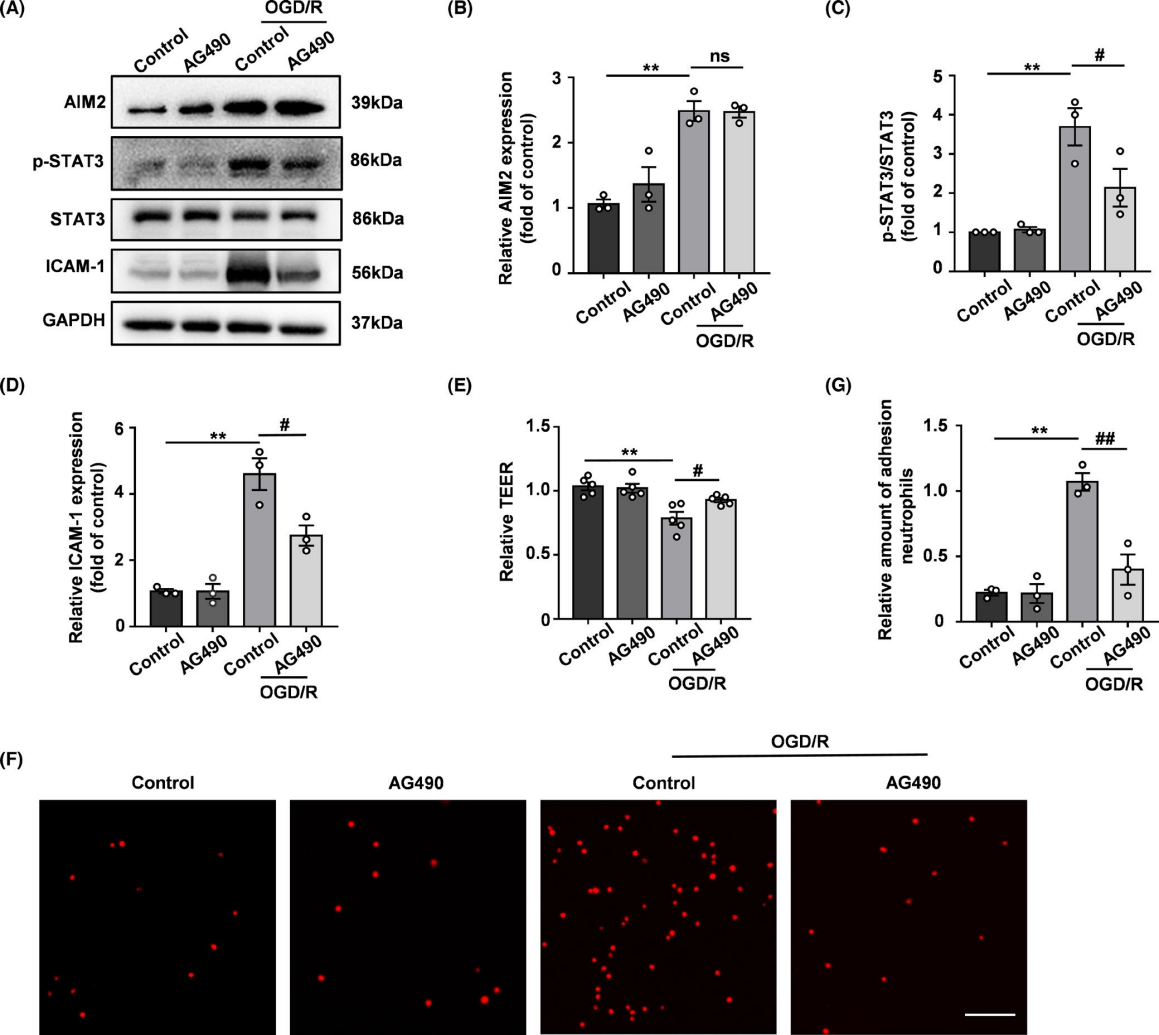
AG490 alleviated OGD/R‐induced BBB breakdown. A, Representative Western blot images of AIM2, p‐STAT3, STAT3, ICAM‐1, and GAPDH expression after OGD/R. B‐D, Quantification of the levels of AIM2, p‐STAT3, and ICAM‐1 normalized to that of GAPDH. E, Quantification of transendothelial permeability as determined by the TEER assay. F, Representative images of neutrophil adhesion to HBMEC monolayers in vitro. Scale bar: 100 μm. G, Quantification of neutrophil adhesion to HBMEC monolayers in vitro. All data are presented as the mean ±SEM. ** *p* < 0.01 compared with the control group. ^#^ *p* < 0.05 and ^##^ *p* < 0.01 compared with the OGD/R‐control group

The selective JAK2/STAT3 inhibitor AG490 was further used to inhibit STAT3 phosphorylation to demonstrate the role of STAT3 in BBB integrity. After 4 h of OGD treatment followed by 20 h of reoxygenation, the levels of p‐STAT3 and ICAM‐1 were significantly increased, accompanied by increased AIM2 expression. However, pretreatment with AG490 reversed the increases in STAT3 phosphorylation and ICAM‐1 expression after OGD/R without affecting the AIM2 level (Figure 7A‐D), indicating that an increased AIM2 expression does not lead to BBB destruction in the presence of AG490. In addition, AG490 treatment increased the TEER and decreased the OGD/R‐induced adhesion of mouse bone marrow neutrophils to HBMEC monolayers (Figure 7E‐G). Considering these results collectively, we conclude that the protective effect of AIM2 deletion is associated with the inhibition of STAT3 phosphorylation.

## 4 DISCUSSION

This study aimed to explore the effects of AIM2 on BBB integrity after ischemic stroke. AIM2^−/−^ and WT mice were subjected to MCAO followed by 24 h and 72 h of reperfusion. Genetic deletion of AIM2 markedly reduced the brain infarct size and BBB damage, as evaluated by EB extravasation and IgG immunostaining assays. The ischemia‐induced loss of TJ proteins was attenuated in AIM2^−/−^ mice. We further discovered that both AIM2 deletion in the MCAO model in vivo and AIM2 knockdown in the OGD/R model in vitro decreased neutrophil infiltration by downregulating ICAM‐1 expression. Moreover, the results revealed that the protective effect on BBB integrity was achieved via the AIM2/STAT3 signaling pathway in endothelial cells. In addition, a STAT3 inhibitor had the same beneficial effect as AIM2 knockdown. In conclusion, our results provide important evidence that AIM2 deletion protects BBB integrity after ischemia or hypoxia by inhibiting STAT3 activation, increasing TJ protein expression, and reducing ICAM‐1 expression.

The BBB is a complex multidimensional reticular barrier that prevents harmful substances from entering the CNS, and cerebral ischemia can disrupt its structure and function. In contrast, BBB disruption exacerbates ischemic stroke outcomes by leading to edema and hemorrhage.^19^ Therefore, identification of an approach to protect against BBB dysfunction in the context of ischemic stroke is urgently needed. Recently, accumulating evidence has shown that the inflammasome is closely associated with BBB damage in many diseases. An in vitro BBB model revealed that blocking caspase‐1 significantly restored all of the contributions to barrier injury, including barrier permeability, TJ protein levels, and peripheral blood mononuclear cell (PBMC) adhesion.^10^ IL‐1 plays a vital role in the pathophysiology of multiple sclerosis (MS), and inhibition of IL‐1 signaling in the BBB markedly ameliorates the severity of MS by downregulating the expression of ICAM‐1 and other proinflammatory mediators.^20^ Moreover, the caspase‐1 inhibitor Ac‐YVAD‐cmk has been demonstrated to alleviate traumatic brain injury (TBI)‐induced BBB damage by suppressing the expression of the pivotal downstream proinflammatory cytokines IL‐1β and IL‐18.^21^ Considering that both caspase‐1 and IL‐1β are key components of the AIM2 inflammasome, we hypothesized that AIM2 plays a crucial role in mediating BBB integrity after ischemic stroke.

HBMECs are the basic components of the BBB and play a critical role in maintaining barrier function. Our previous study showed that AIM2 was expressed in microglia ^15^; however, in the present study, AIM2 was also found to be widely expressed in brain endothelial cells and HBMECs. These findings were consistent with the results of another study on poststroke cognitive impairment in mice.^14^ Moreover, we demonstrated that the AIM2 levels were markedly increased in ischemic brain endothelial cells and HBMECs under hypoxic‐ischemic conditions both in vivo and in vitro. Therefore, AIM2 must play a central role in brain endothelial cells after ischemic stroke.

BBB endothelial cells are connected by TJ proteins, and the mRNA and protein levels of TJ‐associated proteins are significantly decreased after MCAO.^22^ Many drugs that increase TJ protein expression have been indicated to reduce BBB leakage after stroke.^23^, ^24^ During TJ protein disruption, adhesion of neutrophils to endothelial cells can also increase BBB permeability and exacerbate brain injury. Neumann et al. demonstrated that the acute inhibition of neutrophil infiltration into the brain may be a useful method for the clinical treatment of stroke.^25^ In our present study, AIM2 knockout in mice and AIM2 knockdown in vitro improved the BBB structure and function by increasing TJ protein expression and decreasing neutrophil infiltration. However, the mechanism by which AIM2 deletion exerts protective effects remains unclear.

ICAM‐1 is an important endothelial‐associated transmembrane protein that is involved in neutrophil adhesion, and its expression increases dramatically after cerebral ischemia.^26^ The angiotensin‐II receptor inhibitor telmisartan reduced inflammation in the brain by blocking TNF‐α‐induced ICAM‐1 expression and leukocyte adhesion.^27^ A recent study showed that endothelial cell ICAM‐1 expression mediated the migration of T helper (Th) 1 and Th17 effector cells across the BBB in experimental autoimmune encephalomyelitis (EAE).^28^ OGD preconditioning was shown to preserve BBB function during ischemic stress by reducing the ICAM‐1 level.^29^ Chopp et al. revealed that treatment with anti‐ICAM‐1 antibodies selectively reduced the numbers of MPO‐positive and apoptotic cells in MCAO rats.^30^ Therefore, we measured the expression of ICAM‐1 in the context of AIM2 deletion and found that both MCAO and OGD/R increased ICAM‐1 expression, whereas inhibiting AIM2 reversed these effects, suggesting that AIM2 deletion prevents ischemia‐induced BBB injury by downregulating ICAM‐1 expression.

In subsequent experiments, we investigated the mechanisms underlying the beneficial effects of AIM2 on the BBB after ischemic stroke. STAT3 is a crucial transcription factor that controls signal transduction and intercellular communication and activates the transcription of many target genes.^31^ Yun et al. found that microglia‐derived IL‐6 stimulated STAT3 activation in retinal endothelial cells. STAT3 activation increased endothelial permeability by downregulating the expression of ZO‐1 and occludin,^32^ consistent with our results. Previous studies suggested that STAT3 can transcriptionally activate genes associated with vascular barrier integrity, including ICAM‐1 in glioma cells and human aortic endothelial cells (HAECs).^33^, ^34^ Furthermore, AIM2 has been reported to be a central regulator of STAT3. The AIM2 inflammasome prevents intestinal inflammation and dysbiosis by modulating the STAT3 and IL‐18/IL‐22 pathways.^35^ Li et al. found that AIM2 expression was negatively correlated with STAT3 phosphorylation in hypopharyngeal squamous cell carcinoma tissue samples and that patients with both low AIM2 and high p‐STAT3 levels had the worst survival rates.^36^ Based on these findings, we hypothesized that AIM2 deletion preserves BBB integrity by affecting STAT3 activation. In the MCAO model, the p‐STAT3 level was significantly decreased in AIM2^−/−^ mice compared with WT mice. Moreover, AIM2 knockdown attenuated the OGD/R‐induced increase in STAT3 phosphorylation in HBMECs and PBMECs. A recent report suggested that STAT3 inhibition preserves the BBB integrity in OGD/R‐induced in vitro BBB models of rat brain endothelial cells (RBECs).^37^ To further validate the STAT3‐mediated protective effect of AIM2 on the BBB, we also treated HBMECs with a specific STAT3 inhibitor before subjecting them to OGD/R, and AG490 mitigated the effect of AIM2 on BBB breakdown after OGD/R. AG490 alleviated the OGD/R‐induced BBB permeability, and this effect was accompanied by an increased TEER and decreased ICAM‐1 expression and neutrophil adhesion. However, the specific mechanism by which AIM2 affects STAT3 phosphorylation requires further investigation.

Moreover, our study does have some limitations. Firstly, the effects of AIM2 in ischemic stroke were studied in global gene knockout mice. Generating endothelial AIM2 conditional knockout mice may be necessary for further exploration of the cell‐specific mechanism of AIM2 in endothelial cells. Secondly, previous studies from ours and others found that AIM2 is highly expressed in both microglia and endothelial cells in ischemic brain.^14^, ^15^ Microglia are the main component of neurovascular unit, thereby regulating BBB integrity after ischemic stroke.^38^ Suppressing microglia‐mediated inflammatory response or shifting microglia toward an anti‐inflammatory phenotype could alleviate BBB disruption.^39^, ^40^ Our current manuscript focuses on the role of AIM2 in BBB damage, while the crosstalk between microglia and endothelial cells should be discussed in the future.

In summary, our present study suggests that AIM2/STAT3 inhibition in endothelial cells is a promising therapeutic strategy for BBB impairment after cerebral ischemia.

## CONFLICT OF INTEREST

The authors declare that they have no conflicts of interest concerning this article.

## AUTHOR CONTRIBUTIONS

XC conceived, designed, and coordinated the study. S‐yX, H‐jB, SS, and M‐jZ performed the experiments and analyzed the data. S‐nX and YG helped with cells culture and induction of MCAO model. S‐yX, YX, and XC wrote, revised, and checked the data analysis. All the authors revised and approved the final version of the manuscript.

## Supporting information

Figure S1A‐D

Supporting Information

## Data Availability

The data that support the findings of this study are available from the corresponding author upon reasonable request.
