# Supplementary material for: AIM2 deletion enhances blood‐brain barrier integrity in experimental ischemic stroke
Source: CNS Neurosci Ther. 2021 Jun 22;27(10):1224–37. doi: 10.1111/cns.13699 (PMC8446221; doi:10.1111/cns.13699)
Supplement: Supplementary file 1 — Figure S1A‐D [file CNS-27-1224-s002.docx]

supplementary file


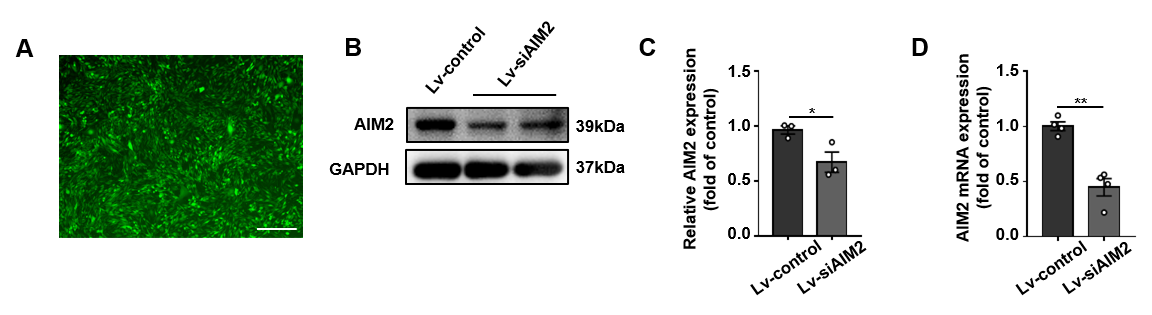


Supplementary figure 1. (A) Immunofluorescence staining after Lv-siAIM2 infection for 3 days. Scale bar: 200 μm. (B) Western blot images indicating AIM2 and GAPDH expression after Lv-siAIM2 or Lv-control infection for 3 days. (C) Quantification of the level of AIM2 normalized to that of GAPDH. (D) AIM2 mRNA expression after infection. All data are presented as the mean ± SEM values. * *p* < 0.05 and ** *p* < 0.01 compared with the control group.
