# Supplementary material for: AIM2 deletion enhances blood‐brain barrier integrity in experimental ischemic stroke
Source: CNS Neurosci Ther. 2021 Jun 22;27(10):1224–37. doi: 10.1111/cns.13699 (PMC8446221; doi:10.1111/cns.13699)

Full unedited gel/blot for Figure 1A

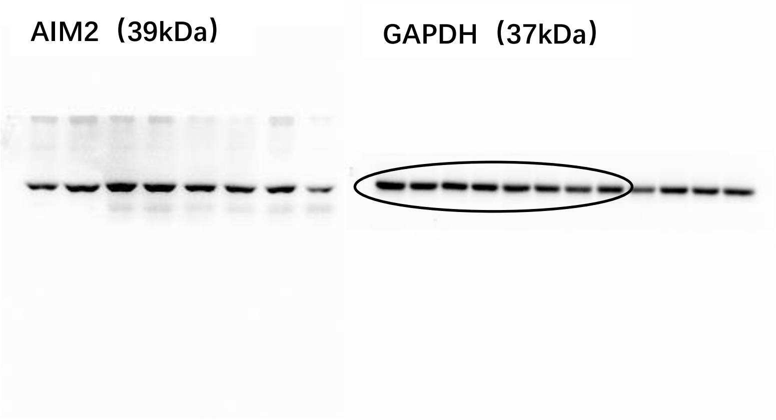

Full unedited gel/blot for Figure 1F

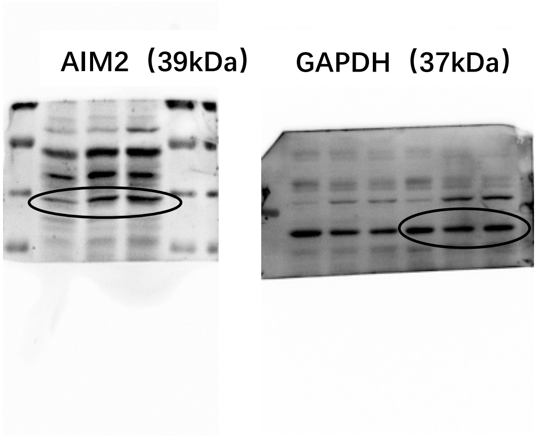

Full unedited gel/blot for Figure 3C

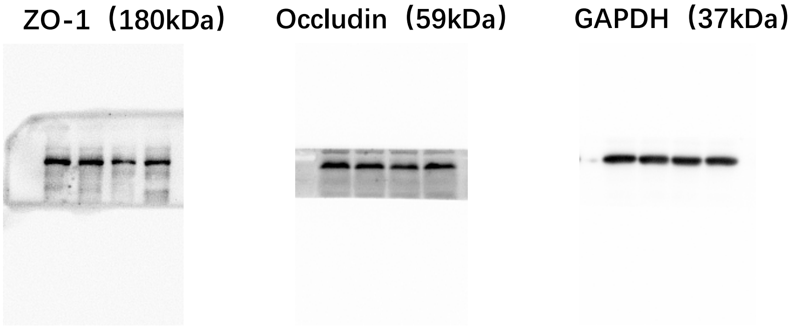

Full unedited gel/blot for Figure 4B

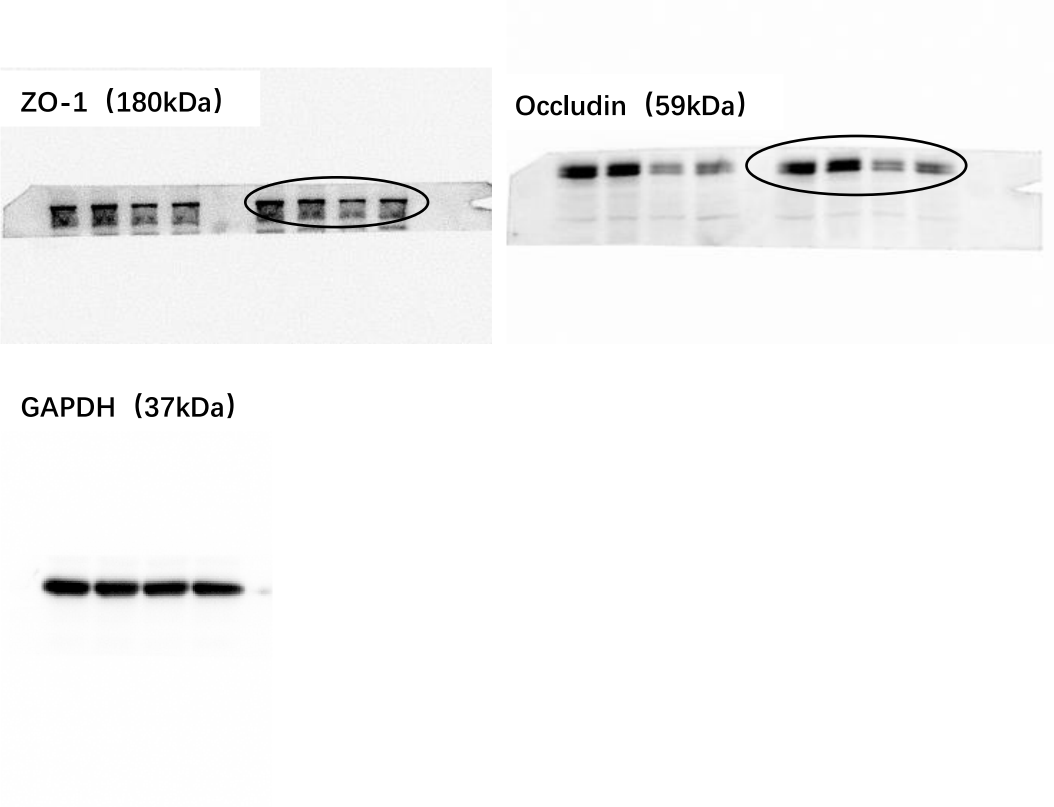

Full unedited gel/blot for Figure 5A

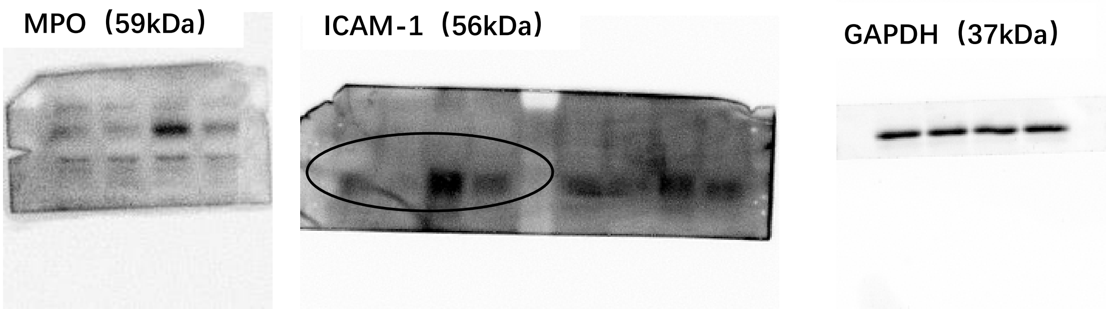

Full unedited gel/blot for Figure 5D

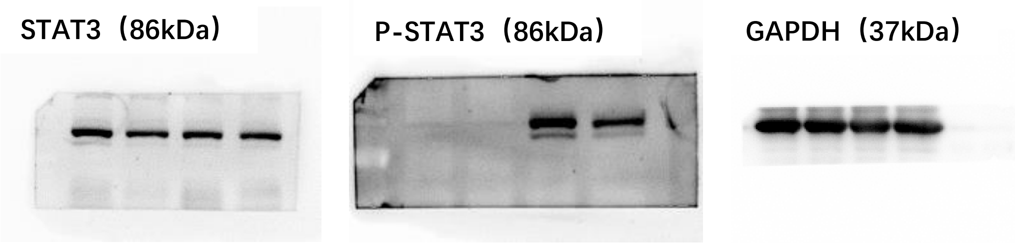

Full unedited gel/blot for Figure 6A

P-STAT3（86kDa）

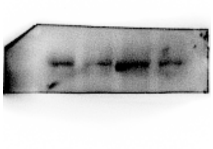

STAT3（86kDa）

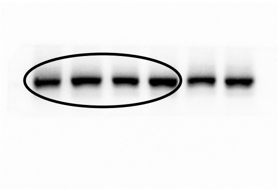

ICAM-1（56kDa）

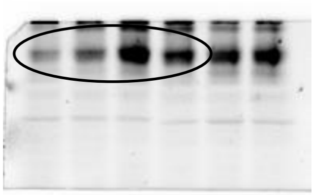

GAPDH（37kDa）

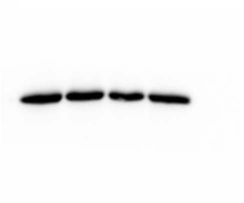

Full unedited gel/blot for Figure 6F

P-STAT3（86kDa）

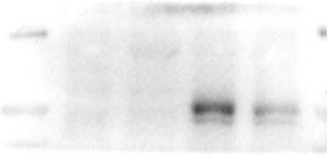

STAT3（86kDa）

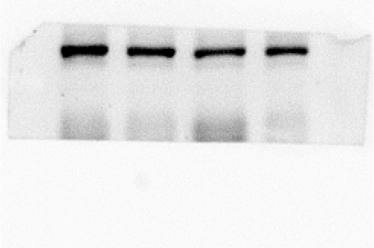

ICAM-1（56kDa）

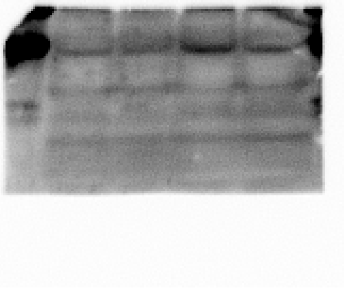

GAPDH（37kDa）

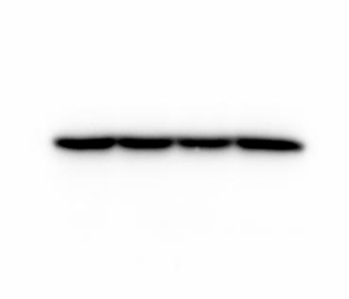

Full unedited gel/blot for Figure 7A

AIM2（39kDa）

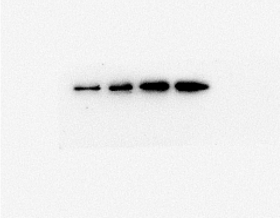

P-STAT3（86kDa）

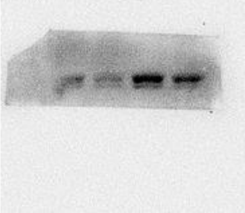

STAT3（86kDa）

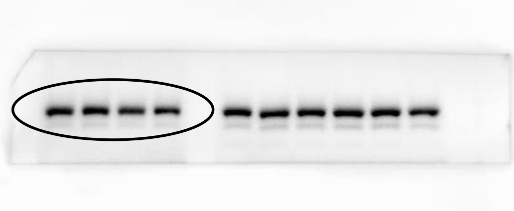

ICAM-1（56kDa）

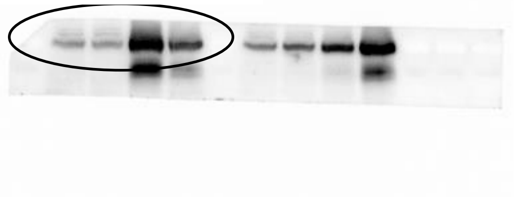

GAPDH（37kDa）

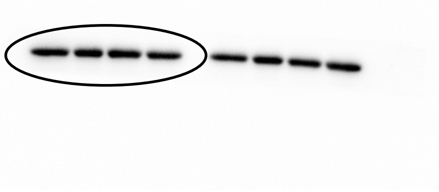

Full unedited gel/blot for Figure S1B

AIM2（39kDa）

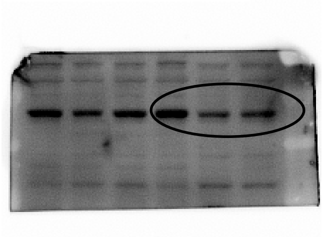

GAPDH（37kDa）

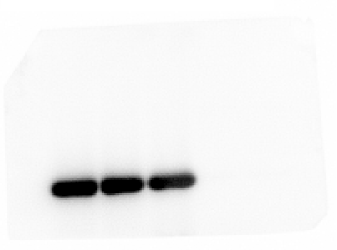

Supplement: Supplementary file 2 — Supporting Information [file CNS-27-1224-s001.pdf]
